# Validation of Electrocardiogram Based Photoplethysmogram Generated Using U-Net Based Generative Adversarial Networks

**DOI:** 10.1007/s41666-023-00156-z

**Published:** 2023-12-01

**Authors:** Jangjay Sohn, Heean Shin, Joonnyong Lee, Hee Chan Kim

**Affiliations:** 1 Institute of Medical & Biological Engineering, Medical Research Center, Seoul National University College of Medicine, Seoul, Korea; 2 Department of Electronic Engineering, Hanyang University, Seoul, Korea; 3 Samsung SDS R&D Center, Seoul, Republic of Korea; 4 Mellowing Factory Co., Ltd., 131 Sapeyong-daero 57-gil, Seocho-gu, Seoul, 06535 Republic of Korea; 5 Interdisciplinary Program in Bioengineering, Seoul National University, Seoul, Korea; 6 Department of Biomedical Engineering, Seoul National University College of Medicine, 103, Daehak-ro, Jongno-gu, Seoul, 03080 Republic of Korea

**Keywords:** Generative Adversarial Networks, Atrial Fibrillation, U-Net, Data Augmentation, Photoplethysmogram, Wearable Healthcare

## Abstract

Photoplethysmogram (PPG) performs an important role in alarming atrial fibrillation (AF). While the importance of PPG is emphasized, there is insufficient amount of openly available atrial fibrillation PPG data. We propose a U-net-based generative adversarial network (GAN) which synthesize PPG from paired electrocardiogram (ECG). To measure the performance of the proposed GAN, we compared the generated PPG to reference PPG in terms of morphology similarity and also examined its influence on AF detection classifier performance. First, morphology was compared using two different metrics against the reference signal: percent root mean square difference (PRD) and Pearson correlation coefficient. The mean PRD and Pearson correlation coefficient were 27% and 0.94, respectively. Heart rate variability (HRV) of the reference AF ECG and the generated PPG were compared as well. The *p*-value of the paired *t*-test was 0.248, indicating that no significant difference was observed between the two HRV values. Second, to validate the generated AF PPG dataset, four different datasets were prepared combining the generated PPG and real AF PPG. Each dataset was used to optimize a classification model while maintaining the same architecture. A test dataset was prepared to test the performance of each optimized model. Subsequently, these datasets were used to test the hypothesis whether the generated data benefits the training of an AF classifier. Comparing the performance metrics of each optimized model, the training dataset consisting of generated and real AF PPG showed a test accuracy result of 0.962, which was close to that of the dataset consisting only of real AF PPG data at 0.961. Furthermore, both models yielded the same F1 score of 0.969. Lastly, using only the generated AF PPG dataset resulted in test accuracy of 0.945, indicating that the trained model was capable of generating valuable AF PPG. Therefore, it can be concluded that the generated AF PPG can be used to augment insufficient data. To summarize, this study proposes a GAN-based method to generate atrial fibrillation PPG that can be used for training atrial fibrillation PPG classification models.

## Introduction

Atrial fibrillation (AF) is the most common arrhythmia caused by chaotic electrical impulses in the atrium [1]. Almost one in four middle-aged persons in the USA and Europe develop AF [2]. Currently, AF affects more than 5.5 million people in the USA; more than 11 million in Europe, and more than 16 million across the Asia Pacific [3]. The conventional method of monitoring AF is to detect abnormal heartbeats using an electrocardiogram (ECG) over a long measurement period [4]. However, AF monitoring through continuous ECG can be challenging because of noncompliance with ambulatory monitoring, allergies to ECG electrodes, and failure to record symptomatic ECG by patients using the ECG device. Therefore, non-intrusive diagnostic devices, termed wearable arrhythmia detection devices, are needed to monitor longer measurement times with reduced compliance issues. Hence, wearable devices that measure photoplethysmography (PPG) have been shown to have the potential to detect AF [5–7] because the pulsatile component of the PPG waveform can extract heart rate, among other cardiovascular parameters [8]. And due to the higher compliance of wearables as well as the possibility of repeat measurements in daily life, the detection of AF using wearables may result in a paradigm shift in the diagnosis of AF [9]. However, research into PPG based detection of AF has been less common than expected, possibly due to the lack of openly available data for PPG measured in AF patients [10]. In a previous study, we have shown that a generative adversarial network (GAN) model can be used to generate PPG using ECG as an input, resulting in PPG characteristics similar to the original PPG measured simultaneously with the input ECG [11]. Although the results in the previous study indicated that the generated PPG followed the heart beat patterns of the source ECG, it lacked clinical applications of the GAN model. Considering that generated PPG follows the heart beats in the ECG, it was hypothesized that arrhythmic beats in AF ECG could be also replicated in the generated PPG. Thus, here, we apply an improved version of our method to generate AF PPG from AF ECG measured in the clinic, and the generated AF PPG is compared to the original AF PPG measured with the ECG. Furthermore, the generated AF PPG data are used to train a PPG-based AF classifier in conjunction with actual AF PPG data to demonstrate the clinical utility of the generated data. The contributions of the present study is that (1) AF PPG can be generated using the proposed GAN model, and (2) generated AF PPG has utility in developing AF detection models using PPG.

## Related Research

There have been other attempts to synthesize synchronized PPG from ECG using GAN methodology [12–14]. While these studies have been successful in reconstructing the trend of the original signal, clinical significance was not demonstrated. [12] used an attention-based generator with multiple discriminators to improve performance, and [13] applied bidirectional grid long short-term memory for the generator, but only proved that the output of the trained networks resulted in similar signals to the original PPG. [14] demonstrated the utility of the network by using the resulting generated PPG as an input to train another AF-detection model, but the contribution of the augmented data was limited. The AF-detection model trained on augmented data resulted in large false positives as compared to the same network trained on real PPG data, undermining the underlying GAN model’s use for medical purposes. In this study, we have addressed these issues by demonstrating our proposed U-Net based GAN model’s ability to generate AF PPG signals which can be used to improve the training of AF detection models.

## Methods

To train the GAN network without bias towards the training dataset, different databases were used to train the network and validate the optimized network. All databases were labeled by expert cardiologists. The Seoul National University Hospital Institutional Review Board (IRB) approved the IRB exemption for this study (IRB exemption:2012-064-1180).

### Dataset for Training AF PPG Generation Model

To train the proposed model, public biosignal data from 6388 patients undergoing various surgeries at the Seoul National University Hospital (VitalDB), collected using the Vital Recorder program, were used [15]. Lead II ECG and finger PPG were recorded at 500 Hz on a commercial patient monitor (Tram-Rac4A; GE, Milwaukee, WI, USA). The signals were down sampled to 100 Hz. Out of the 6388 individual patients in the database, 6147 recordings with simultaneously measured ECG and PPG were used. After detecting the R-peaks of ECG using the Pan–Tompkins algorithm [16] and systolic peaks of PPG using the findpeaks function from the MATLAB signal toolbox, recordings with correlation coefficients of above 0.96 between the extracted peak arrays were selected. On average, each recording was 93 min long; however, only regions with valid signal ranges were selected for further processing. After comparing the R-R interval and interval between the PPG peaks, the signal for analysis was selected only when it had a correlation coefficient above 0.96. All other cases were excluded from the analysis, and a total of 5253 recordings remained.

### Proposed GAN Architecture

Generative adversarial networks are widely used owing to their outstanding performance in synthesizing signals or images [17, 18]. Attempts have been made to synthesize PPG data using an applied GAN [11, 12, 19]. However, because of the random seeding of GAN to produce the resulting PPG, the resulting PPG contains randomly distributed characteristics, such as heart rate [19], and therefore may not have significant clinical value [12].

**Fig. 1:**
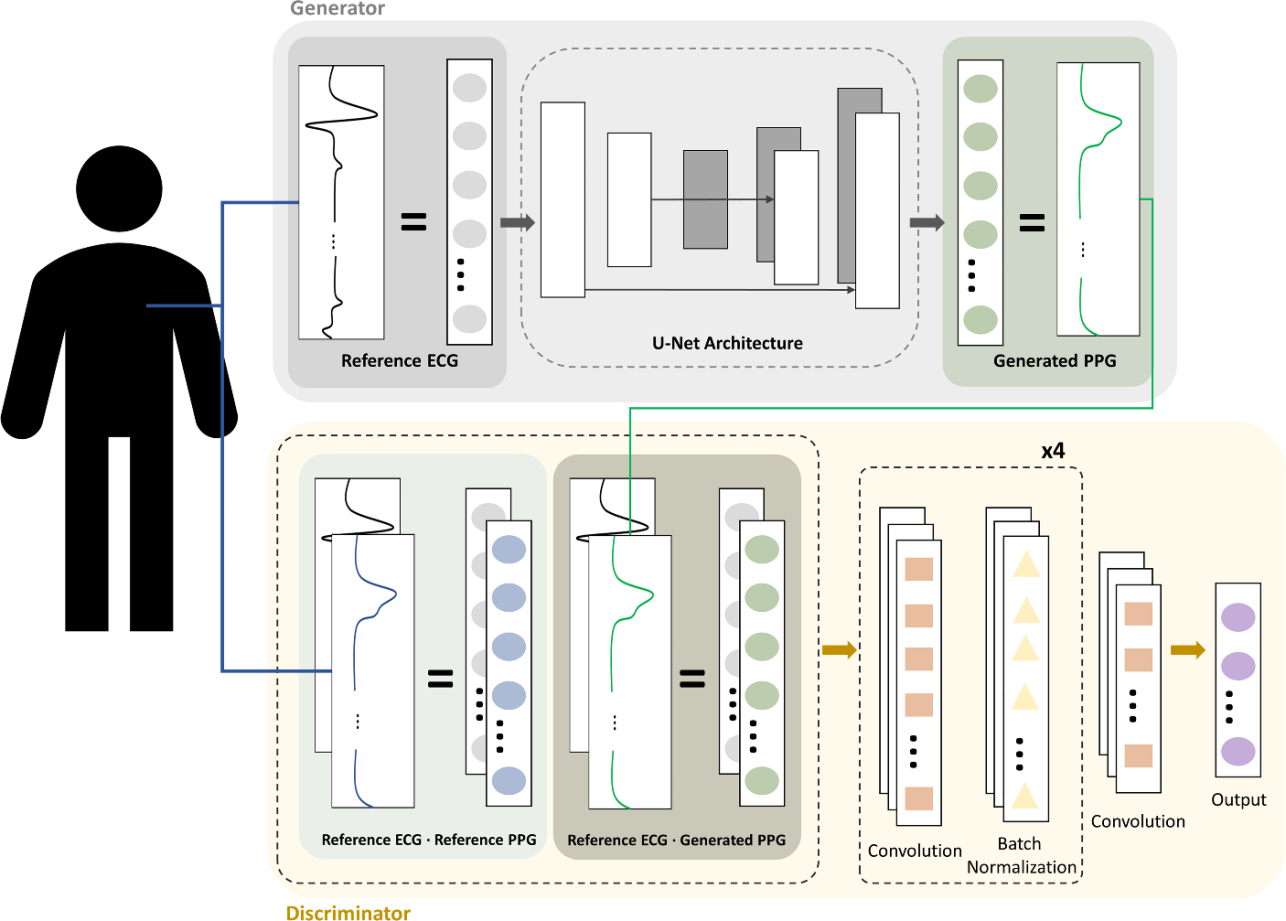
Flow diagram for the proposed GAN architecture. The U-Net-based generator takes ECG as an input, and outputs the generated PPG. The output from the generator is fed into the CNN-based discriminator which is trained to distinguish between synthetic PPG and real PPG. [GAN: generative adversarial networks, ECG: electrocardiogram, PPG: photoplethysmogram, CNN: convolutional neural network]

To overcome these limitations, the proposed model uses concepts related to image translation. The network architecture is described in Fig. 1, where the generator takes an ECG segment as an input to generate a synthetic PPG segment. Subsequently, the concatenated ECG and PPG are passed to the discriminator to distinguish between synthetic and actual PPGs.

A convolution-based U-Net architecture was used for the generator, and a convolution network was used for the discriminator, as shown in Fig. 1. The U-Net architecture comprises of encoder and decoder parts, wherein the encoder compresses the ECG signal using several convolution layers. The core representation of the input signal is extracted at the end of the encoder. Subsequently, the decoding section of the network uses the compressed information to generate a matching PPG signal. In addition, skip connections were added between the encoding and decoding layers to compensate for the loss of locational information during the encoding procedure.

**Fig. 2:**
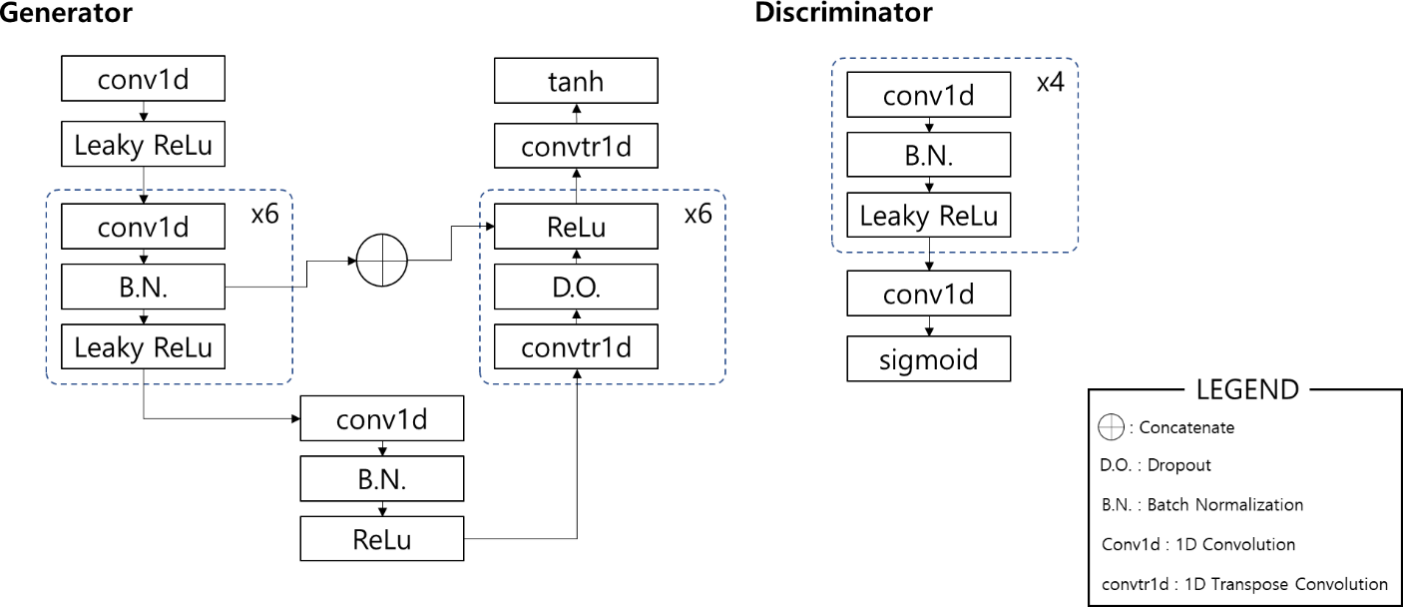
Description of generator and discriminator network. The generator uses a U-Net-based architecture, and the discriminator is a Markovian discriminator

Following the generation, the discriminator is presented with real and generated PPG data, and trained to distinguish the input as real or synthetic. Adding the discriminator improves the generator model, enhancing output details after optimization.

### Validation of Generated AF PPG for Classification Model Development

**Fig. 3:**
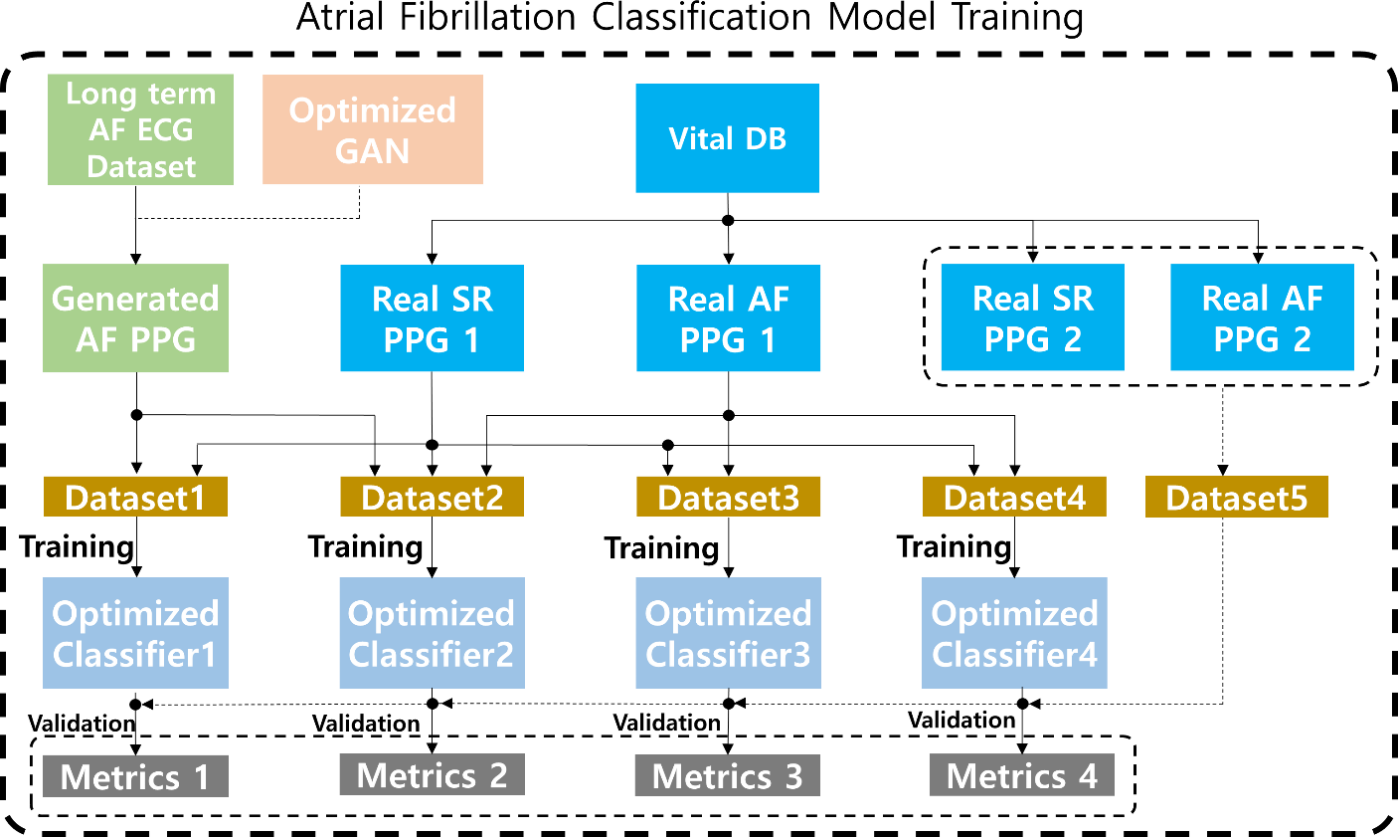
The overall process of data distribution. The four different datasets were prepared to optimize the AF classification model with the same structure. The remaining dataset was used to validate the optimized classification model. The performance metrics, such as accuracy were compared between the optimized models. [SR: Sinus Rhythm, AF: Atrial Fibrillation]

To validate whether the generated AF PPG segments can be used to improve PPG-based AF classification, the generated segments were used to train a simple convolution-based PPG AF classification model. Datasets with different ratios of the generated and real datasets were created to train and validate the classification model. The optimization accuracy of the AF classification was compared between the datasets, as shown in Fig. 3 and Table 1.

**Table 1:** The table of AUROC for the datasets and 95% confidence interval. [AUROC: area under the receiver operating characteristic curve]

Dataset	Accuracy	AUROC[CI]	Precision	Recall	F1 Score
1(Generated)	0.945	0.980 [0.978–0.981]	**0.957**	0.953	0.955
2(Mixed)	**0.962**	0.986[0.985–0.988]	0.949	**0.99**	**0.969**
3(Real)	0.961	**0.988[**0.986–0.989]	0.951	0.988	**0.969**
4(Real Half)	0.891	0.947[0.944–0.951]	0.867	0.970	0.914

Five types of datasets were used here as seen in Table 2. The first dataset was generated PPG. To generate the synthetic AF PPG dataset, the optimized GAN was used along with an AF ECG database (Long Term AF Database v1.0.0) [20, 21]. The second and third datasets included real sinus rhythm PPG and real AF PPG data. The fourth and fifth datasets were obtained from the VitalDB (separate from the second and third datasets), which was used to validate the effectiveness of the generated data.

**Table 2:** Summary of the characteristics of the five different datasets used in training and testing the PPG AF classifier

Dataset	Source (# of subjects)	Data Types	Purpose	Number of samples
1	Long Term AF DB (AF patients 45) VitalDB (Normal 45)	Real ECG, Generated PPG	Training	Generated AF : 11,550 Normal : 11,550
2	Long Term AF DB (AF patients 23) VitalDB (AF patients 22, Normal patients 45)	Real ECG, Generated PPG, Real PPG	Training	Generated AF : 5775 Real AF : 5775 Real Normal : 11,550
3	VitalDB (AF patients 45, Normal patients 45)	Real ECG, Real PPG	Training	Real AF : 11,550 Real Normal : 11,550
4	VitalDB (AF patients 23, Normal patients 23)	Real ECG, Real PPG	Training	Real AF : 5775 Real Normal : 5775
5	VitalDB (AF patients 53, Normal patients 27)	Real ECG, Real PPG	Testing	Real AF : 11,970 Real Normal : 7590

The second and third datasets comprised 770 real 30-s AF PPG and SR PPG segments, respectively, which were obtained from VitalDB and annotated by an expert cardiologist. Using a custom graphic user interface for annotation and following the American Heart Association Guideline for the Management of Patients with Atrial Fibrillation [22], a single cardiologist labeled the AF events in the presented datasets. Subsequently, each 30-second segment was sliced into 15 of 15-second segments translated by 1 second. Thus, a total of 11,550 15-second real AF PPG segments were collected. The Long Term AF Database v1.0.0 consists of 84 long-term paroxysmal or sustained AF ECG recordings sampled at 128 Hz. From the 84 ECG recordings, 59 recordings which had more than 2 AF rhythms beats were included in this study. From the 59 recordings, 3 recordings with ECG too noisy for peak detection were excluded, based on the detection of average heart rate less than 30 beats per minute. Then, the recordings were down-sampled to 100 Hz, and the AF-annotated parts of the recording were segmented into 30-second without overlap. These 30-second ECG segments were then used to generate 30-second PPG segments. After the generation of the synthetic data, in order to match the number of segments between the real and the generated PPG, 770 segments were randomly selected for matching the amount of data that can be extracted from VitalDB, and the same slicing method was applied to generate 11,550 15-second synthetic AF PPG segments.

Lastly, fourth and fifth datasets were used to validate the performance of the optimized AF classification models. These were composed of 790 real 30-second AF PPG and 506 SR PPG segments respectively, and were obtained from VitalDB. Augmentation was performed in the same way as described above, resulting in 11,970 of 15-second real AF segments and 7590 of 15-second sinus rhythm segments.

**Fig. 4:**
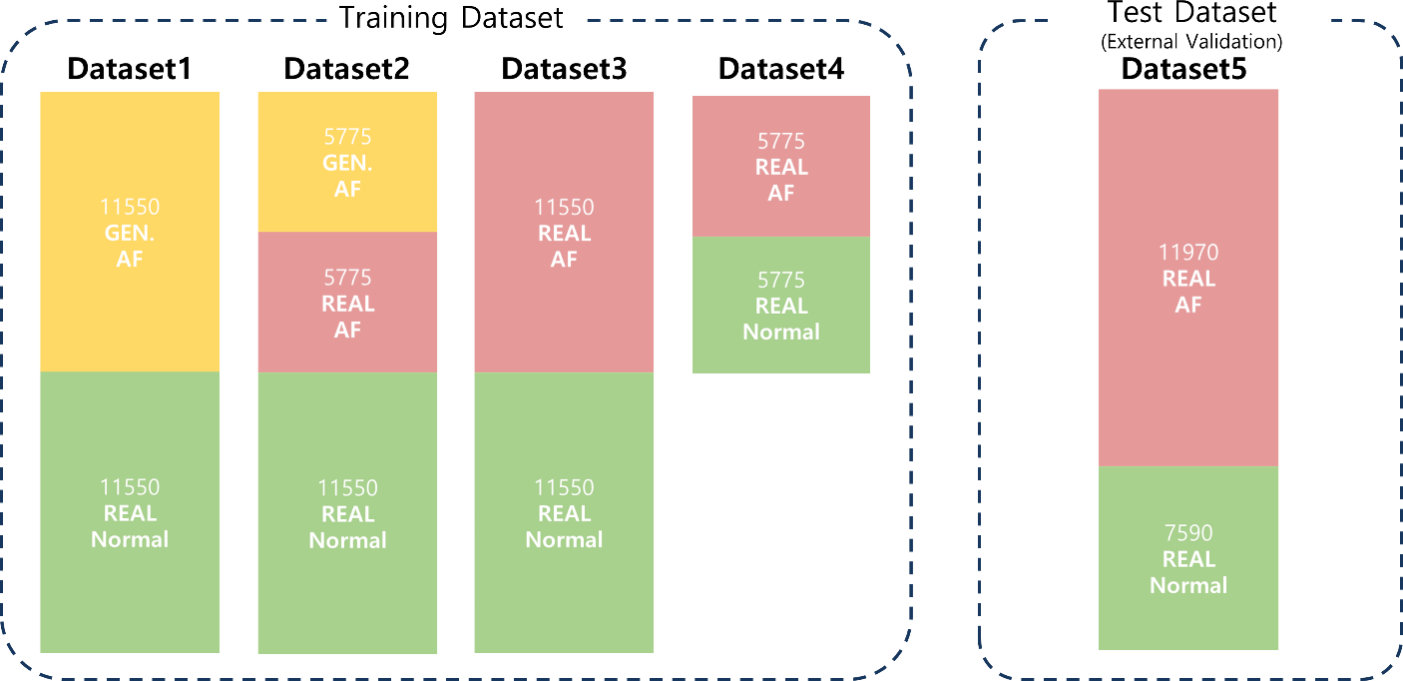
The details of constructed training datasets. The number written on each block indicates the number of 15-second segments

The first four datasets above were combined into four training datasets. For Dataset 1, all generated data were used were used with 11,550 sinus rhythm PPG segments. For Dataset 2, half of the generated AF PPG (5775 segments) and half of the real AF PPG (5775 segments) were used with 11,550 sinus rhythm PPG segments. For Dataset 3, all real AF segments (11,550 segments) were used with the same number of sinus rhythm PPG segments. For Dataset 4, half of the real AF segments (5775 segments) and half of the real sinus rhythm segments (5775 segments) were used. Furthermore, a test dataset exclusive from training or validation data was formed from 11,970 of 15-second real AF segments and 7590 of 15-second sinus rhythm segments. The details of the datasets are shown in Fig. 4.

### Classification Model Architecture

A CNN-based classification model was constructed. The input to the model was formed of a 1500-length input layer corresponding to 15-second segment sampled at 100 Hz. The classifier consisted of a 6-layer CNN, with 4, 8, 16, 32, 64 and 128 filters. Kernel sizes of 3 were used for each layer. The kernel weights were initialized using a truncated normal initializer with a standard deviation of 0.05 and the biases were initialized with 0. After each convolution, the hidden states were activated via a leaky rectified linear unit (ReLu) with an alpha value of 0.15. In the final layer, the hidden states were global averaged and fully connected into an output layer with softmax activation for classification. The final output represents probability of each class, where class 0 is normal sinus rhythm PPG and class 1 is AF. Cross-entropy was used as the loss function and Adam optimizer was used with a learning rate of 0.001. To determine the classification accuracies of the trained models, AUROC and confusion matrix were calculated for each dataset following optimization.

### Evaluation of Generated Dataset Using ***t-SNE***

*t*-distributed stochastic neighborhood embedding (*t*-SNE) introduced by van der Maaten and Hinton in 2008 [23] was used for visualizing high-dimensional data using effective nonlinear dimensionality reduction.

*d*-dimensional input dataset by $\mathcal{X}=\{{x}_{1},{x}_{2},\dots ,{x}_{n}\}\subset {\mathbb{R}}^{d}$ is embedded to *s*-dimensional output dataset, denoted by $\mathcal{Y}=\{{y}_{1},{y}_{2},\dots ,{y}_{n}\}\subset {\mathbb{R}}^{s}$, where $s\ll d$ and the most common value for and usually *s* is 2 or 3 for visualization purposes. The joint probability ${p}_{ij}$ measuring the similarity between ${x}_{i}$ and ${x}_{j}$ in high dimensions, is calculated as followed

$${p}_{j|i}=\frac{{e}^{-\frac{{\parallel {x}_{i}-{x}_{j}\parallel }^{2}}{2{{\sigma }_{i}}^{2}}}}{\sum _{k\ne i}{e}^{-\frac{{\parallel {x}_{i}-{x}_{k}\parallel }^{2}}{2{{\sigma }_{i}}^{2}}}}$$

and

$${p}_{ij}=\frac{{p}_{i|j}+{p}_{j|i}}{2n}$$

The similarity between points ${y}_{i}$ and ${y}_{j}$ in low dimension is computed as

$${q}_{ij}=\frac{{(1+{\parallel {y}_{i}-{y}_{j}\parallel }^{2})}^{-1}}{\sum _{k\ne l}{(1+{\parallel {y}_{k}-{y}_{l}\parallel }^{2})}^{-1}}$$

*t-*SNE determines the points $\{{y}_{1},{y}_{2},\dots ,{y}_{n}\}$ that minimize the distribution difference between the joint distribution *P* and *Q* measured by the Kullback-Leibler divergence as followed

$$C\left(\mathcal{Y}\right)=KL\left(P\parallel Q\right)=\sum _{i\ne j}{p}_{ij}\text{l}\text{o}\text{g}\frac{{p}_{ij}}{{q}_{ij}}$$

First, the points $\mathcal{Y}$ are initialized randomly, and the cost function $C\left(\mathcal{Y}\right)$ is minimized using gradient descent. The gradient can be represented as

$$\frac{\partial C}{\partial {y}_{i}}=4\sum _{j\ne i}({p}_{ij}-{q}_{ij}){q}_{ij}Z({y}_{i}-{y}_{j})$$

where $Z$ is a global normalization constant expressed as

$$Z=\sum _{k\ne l}{(1+{\parallel {y}_{k}-{y}_{l}\parallel }^{2})}^{-1}$$

Probability density estimation was used to measure the overlap between the two *t-*SNE distributions, for which, a normal kernel function was selected. It is assumed that the probability density function of the generated AF PPG *t-*SNE distribution is ${P}_{1}$, and that of the real AF PPG *t-*SNE distribution is ${P}_{2}$. If ${P}_{1}$ and ${P}_{2}$ perfectly overlap, the two have the same distribution and the equation below yields a value of 1. In contrast, if the two distributions are perfectly separated from each other, value 0 would be obtained.

$$Degree\,of\,overlap\left( {DO} \right) = \sum {\sqrt {{P_1} \times {P_2}} }$$

Thus, if the two distributions are similar, the value would be closer to 1, and for the opposite case, the value would be closer to 0.

## Result

### Morphology Comparison Result

**Fig. 5:**
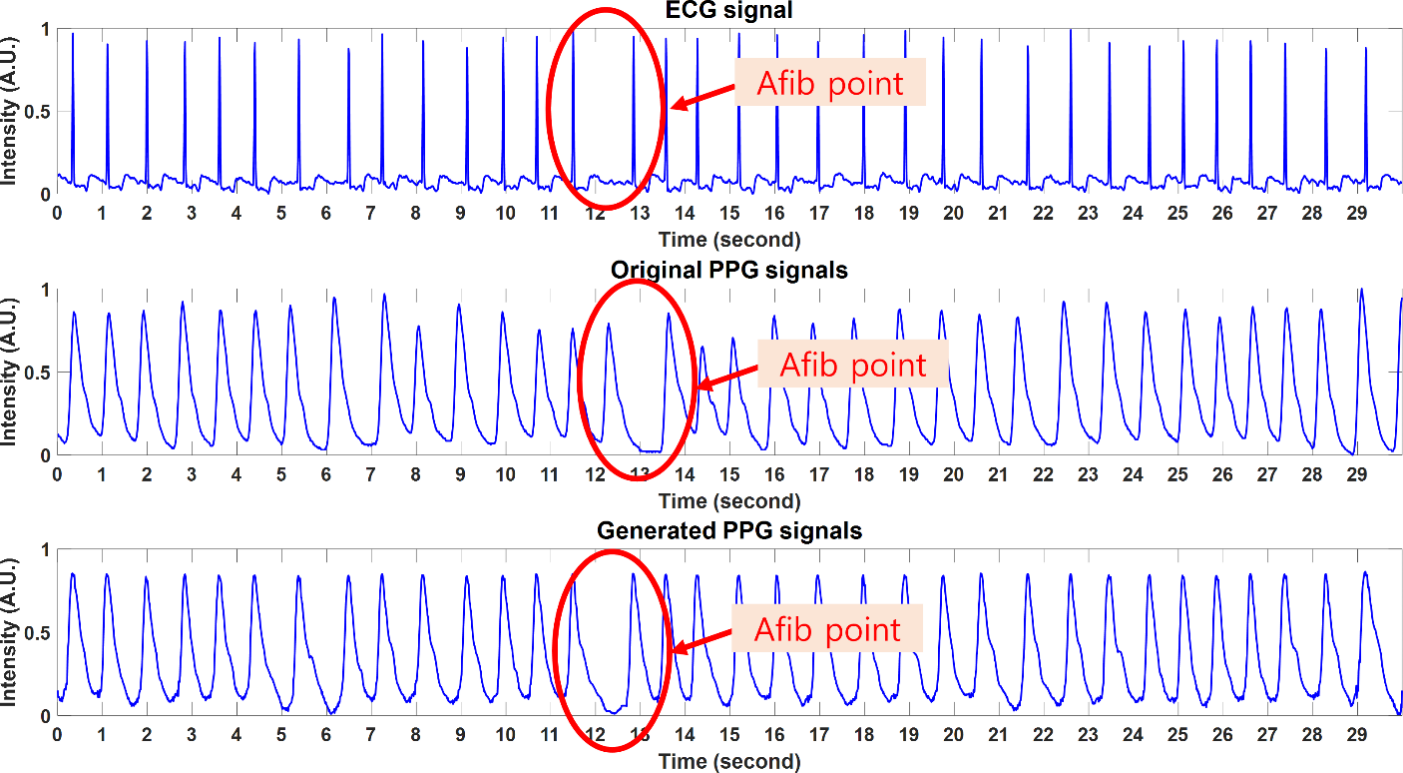
(**a**) Input ECG example. (**b**) Generated PPG and original PPG examples. [ECG: electrocardiogram, PPG: photoplethysmogram]

An example of a simultaneously measured ECG-PPG pair and the corresponding GAN-generated PPG is shown in Fig. 5.

The comparison between beat-by-beat heart rates extracted from reference AF ECG and synthetic AF PPG using paired *t*-test resulted in a *p*-value of 0.248 and correlation coefficient of 0.94 ± 0.14. The results of calculated Percent Root mean square Difference (PRD) and Pearson correlation coefficient between the reference PPG and synthetic PPG are summarized in Table 3.

**Table 3:** PRD and Pearson correlation coefficient calculated between reference and synthetic PPG using the test dataset. [PRD: percent root mean square difference]

Metric	Total Number of Segments	Total Number of Outliers	Mean	STD
PRD(%)	1893	23	26.8	7.3
CC		102	0.940	0.045

### Effect on AF Detection Classification Accuracy

All models resulted in AUROC higher than 0.9. The highest AUROC value of 0.988 was obtained by the model trained with Dataset 3, which consists of real PPG segments for both AF and sinus rhythm recordings. The lowest performance was obtained by the model trained with Dataset 4, which consists of only half of the real data. The AUROC values and 95% confidence intervals for each model are shown in Table 3.

**Fig. 6:**
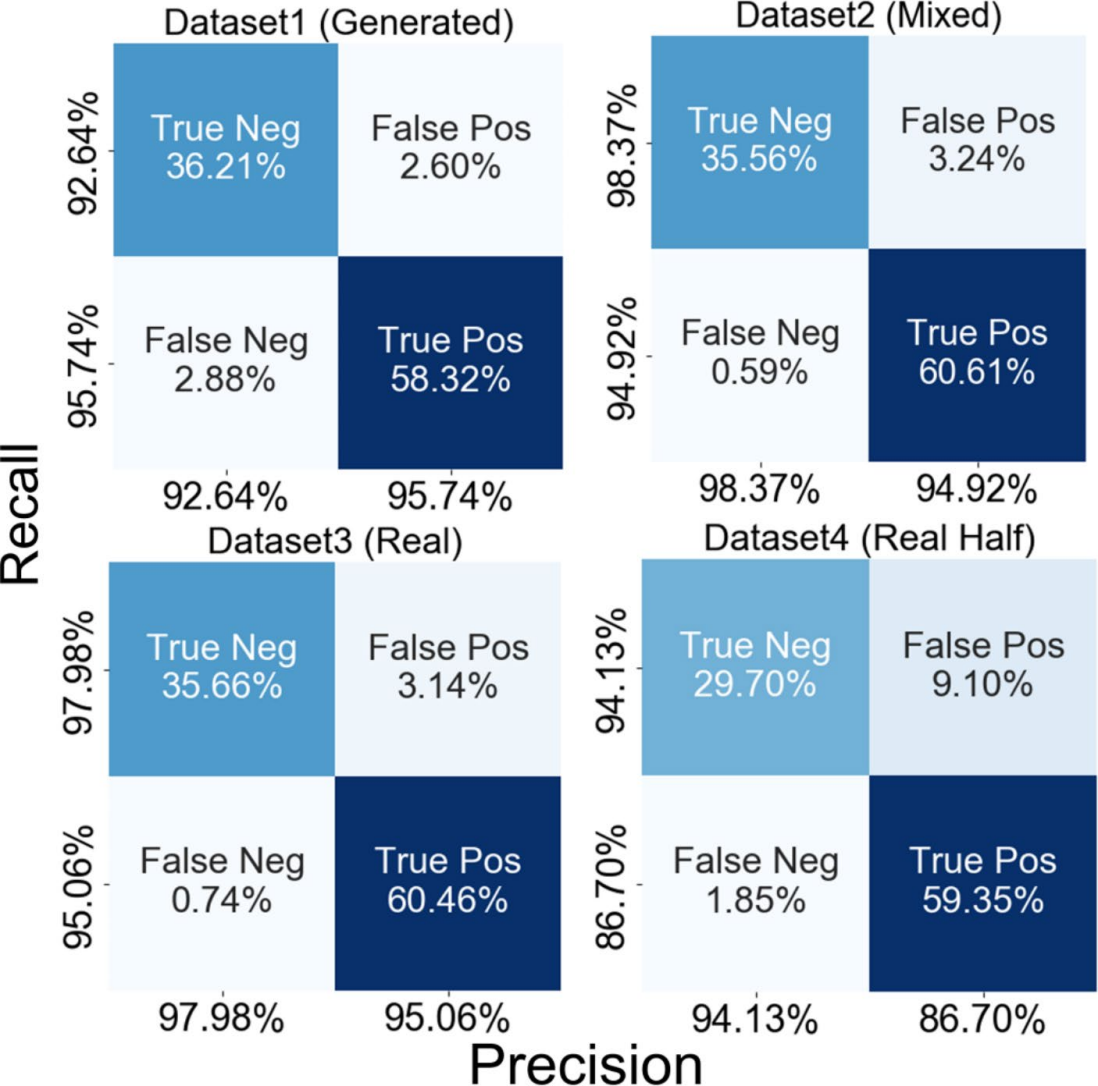
The confusion matrices of AF classification trained using each dataset.

Confusion matrices for each model are shown in Fig. 6, along with calculated performance metrics. Dataset 2 and Dataset 3 yielded similar accuracies with the same F1 score value of 0.969. Dataset 1, consisting of only generated AF PPG, showed slightly lower values, while Dataset 4 showed the lowest performance metric values and AUROC.

### t-SNE and DO Value Comparison

**Fig. 7:**
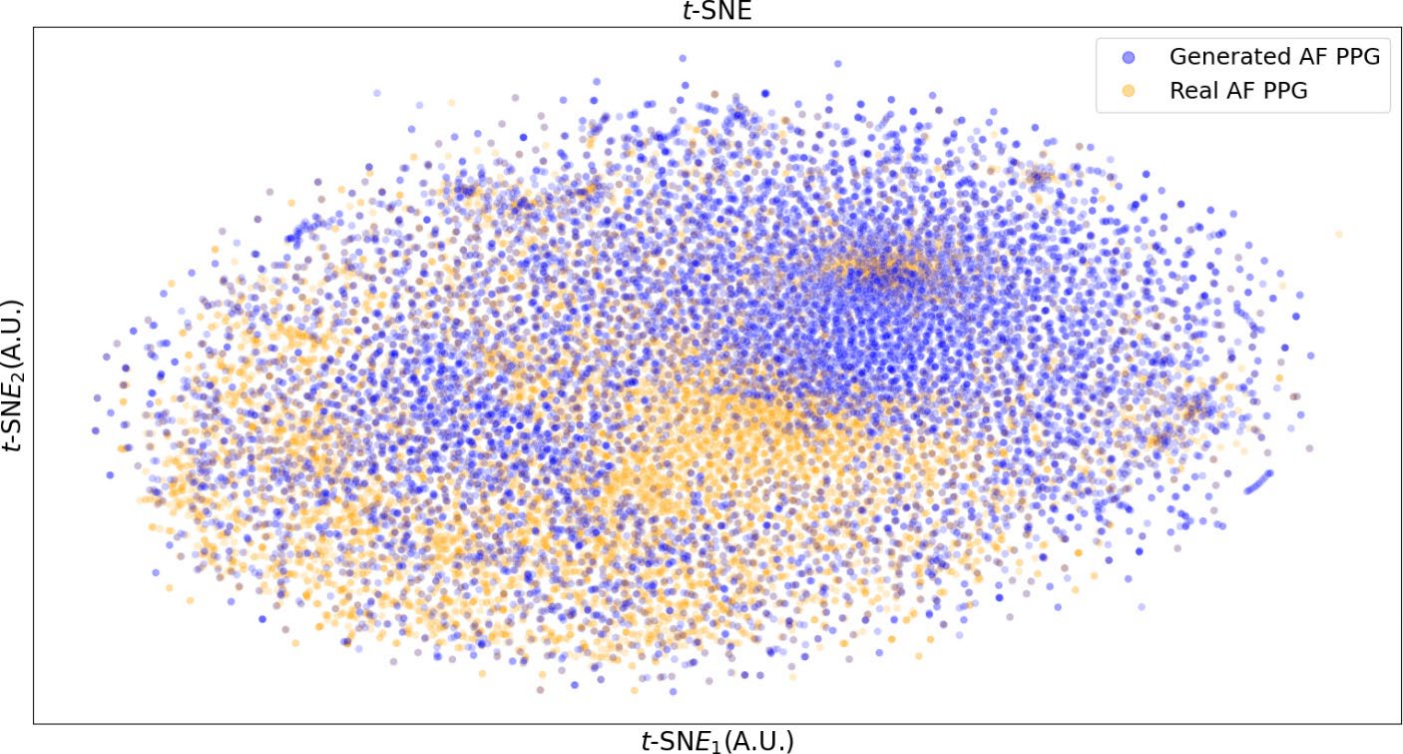
Data distribution of real AF PPG and generated AF PPG observed on a 2D plot obtained using the t-SNE method. [AF: atrial fibrillation, PPG: photoplethysmogram].

**Fig. 8:**
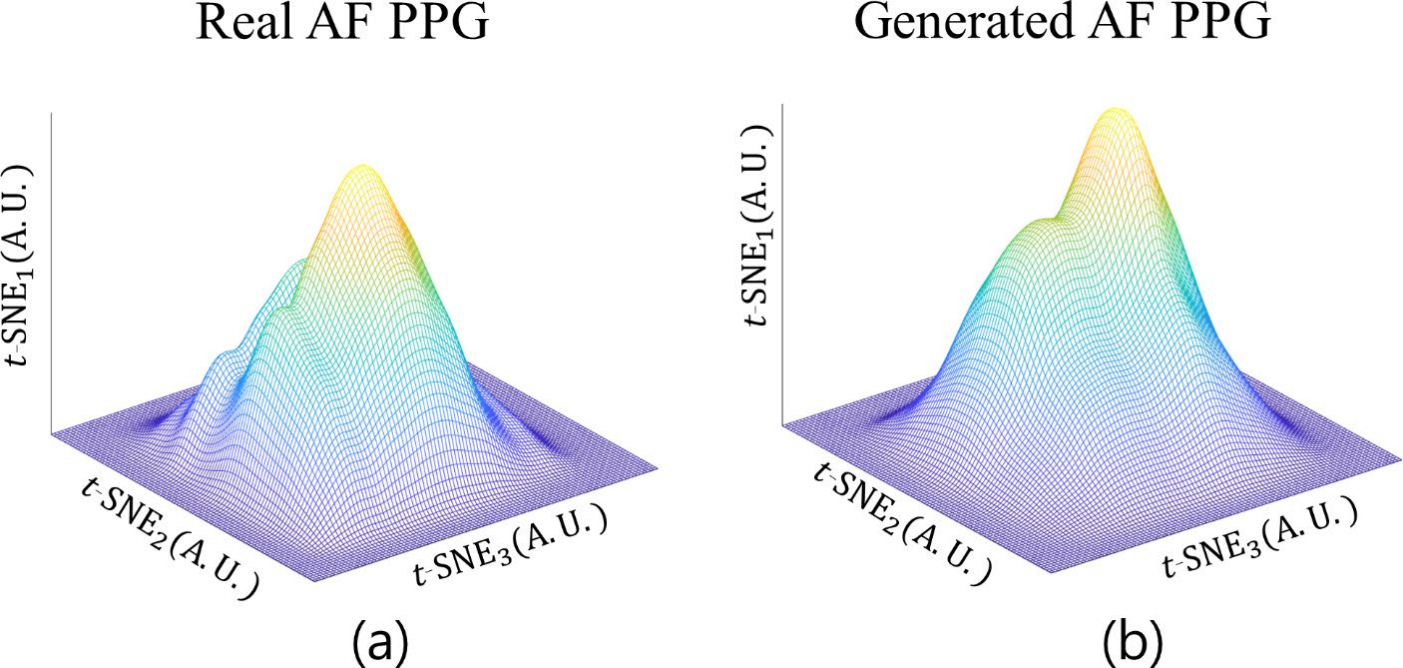
(**a**) Estimated probability density function of t-SNE for the real AF PPG. (**b**) Estimated probability density function of t-SNE for the generated AF PPG [AF: atrial fibrillation, PPG: photoplethysmogram]

**Fig. 9:**
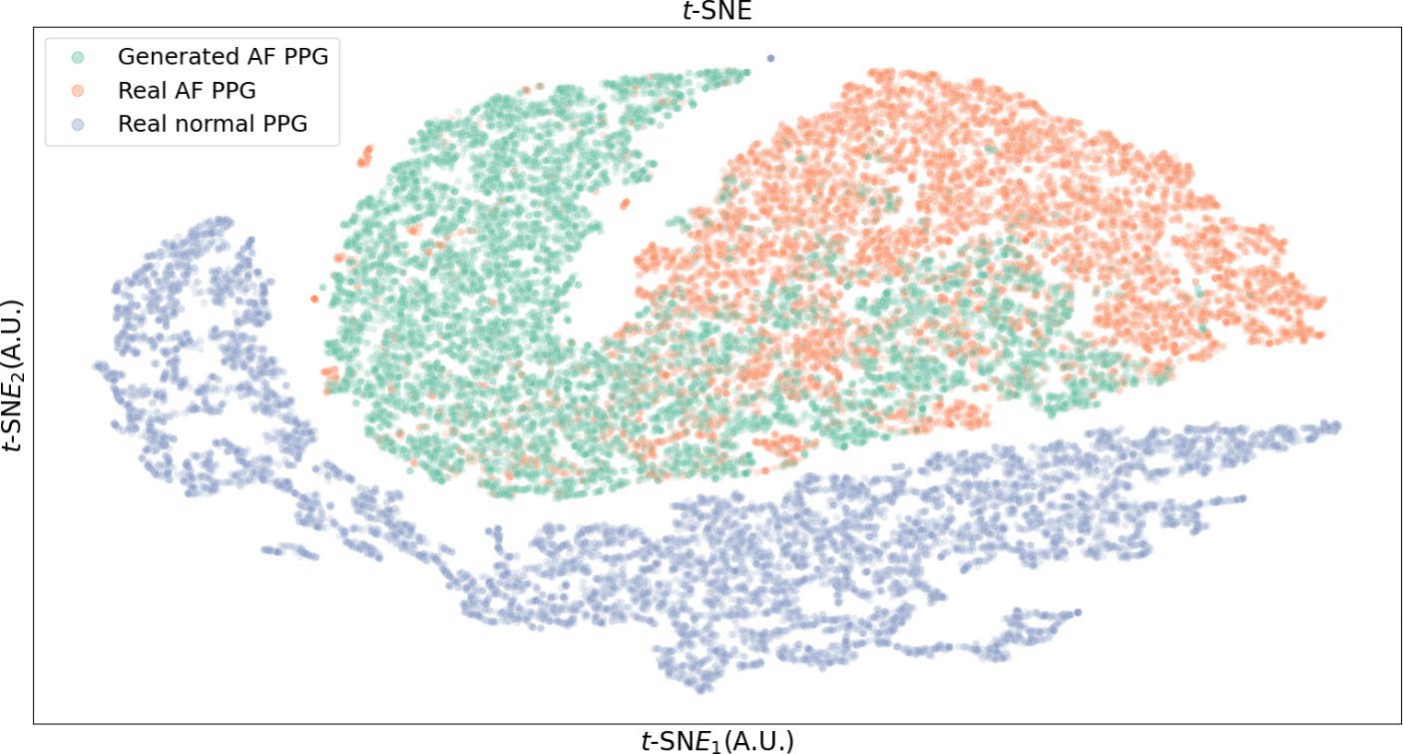
Data distribution of last feature map of the AF classification model observed on a 2D plot obtained using the t-SNE method. Green dot represents the generated AF data, orange dot represents the real AF data, and blue dot represents the real sinus rhythm PPG. [AF: atrial fibrillation, PPG: photoplethysmogram]

As shown in Fig. 7, the generated AF PPG and real AF PPG show high degree of overlap. Figure 8 shows the estimated probability density of *t*-SNE distributions of both datasets with DO value at 0.937. The *t-*SNE results of the final feature map extracted from the classification model are shown in Fig. 9.

## Discussion

### Heart Rate Preservation in Generated PPG and Applications to Data Augmentation

The correlation coefficient between the beat-by-beat heart rate measured across the atrial fibrillation ECG segments and in the corresponding generated PPG segments was 0.94 ± 0.14, and comparison using paired *t*-test yielded a *p*-value of 0.248, indicating the highest association with no significant difference between the variability measured in the two signals. These preliminary results indicate that the proposed GAN model could potentially be used for generating atrial fibrillation PPG data using open atrial fibrillation ECG datasets. Therefore, it is possible that the proposed GAN can be used to improve the accuracy of PPG-based atrial fibrillation detection, with the augmentation of PPG data reflecting inter-beat interval variability of atrial fibrillation ECG.

### Application of Generated PPG Data in AF Classification Task

In order to test the utility of the generated AF PPG, the generated PPG was mixed into real AF PPG data and sliced into 4 different datasets. By comparing the results between Dataset 2, where the generated AF dataset is used for augmentation, and Dataset 4, it was shown that the generated AF PPG can be used to improve the training of the classification model. All metrics for classification improved between Dataset 4 and Dataset 2, showing that GAN-generated PPG segments can better optimize the classification model. Furthermore, removing generated AF data from Dataset 2 resulted in decreased model accuracy to 0.956, while Dataset 3, with double the number of real AF data achieved high accuracy, and Dataset 4, achieved lower accuracy with less available data, indicating that the absolute number of data available can also affect model performance. Thus, in case of data insufficiency, GAN-generated AF PPG segments may be used as viable substitutes for real AF PPG segments.

In order to test the generated AF PPG data on optimizing AF classification without real AF PPG data, Dataset 1 was created. Although the performance metrics for the Dataset 1 model were relatively lower than those of the Dataset 3 model, the absolute values indicate that this dataset can also be used to train an AF PPG classification model. Moreover, considering that the test dataset was made up of data from the VitalDB, the model trained on Dataset 3 is expected to outperform the Dataset 1 model, as Dataset 3 composes of data from VitalDB while Dataset 1 composes of data generated from the Long Term ECG database. Thus, it is possible that switching the external test dataset to an alternative other than VitalDB may result in closer performance metrics between the Dataset 1 model and the Dataset 3 model.

### Data Analysis Using ***t-***SNE

*t-*SNE was applied to the GAN-generated AF signals and the real AF signals. As shown in Fig. 7, the 2-dimensional representation of both signals overlap, which indicates that the generated signals are similar to the real signal. Figure 9 shows the *t-*SNE of the feature map extracted from the classification model at the last layer prior to softmax activation. As shown, normal PPG is distinct from the AF PPG, but, the generated AF and the real AF PPG overlap, further validating that the generated AF PPG is closer in morphology to real AF PPG rather than sinus rhythm PPG.

However, there is a time delay between the original ECG and PPG called Pulse Transit Time (PTT). The variety of PTT range is widely distributed based on subject physiology. Although PTT isn’t recreated in the generated PPG as shown in Fig. 5, PTT did not affect accuracy of the AF classification model during validation.

### Application of the Proposed GAN for Augmenting Datasets for PPG Arrhythmia Classification Training

Currently, most reported works of PPG based AF detection use datasets containing paroxysmal AF and sinus rhythm signals rather than a mixture of different AF types. The synthetic dataset would be balanced in the number of sinus rhythm signals and with various different types of AF, without one dominating class, which would be suitable for training deep learning models for AF classification.

Although PPG can be acquired using a simple setup, the most critical limitation of PPG is the motion artifact. PPG signals with motion artifacts can have similar characteristics to AF PPG signals, such as irregular pulse-to-pulse intervals, which could result in a false positive for AF detection [24]. In one study, it was reported that 40% of the collected PPG signals were unreliable for determining the presence of AF due to motion artifacts [4]. Considering that ECG measurement tends to be more stable during motion, the proposed method may be more robust in generating training data for AF PPG. Applying an AF classification model trained on such dataset would require only using stable data measured using wrist PPG, but the value of such a method is yet to be determined in relation to AF detection.

Another problem with creating an AF PPG dataset is that the data needs to be manually annotated, which is time-consuming and resource-intensive. In particular, the annotation of AF PPG requires simultaneous measurement of the ECG for verification of AF. However, this the method described in this study used pre-annotated AF ECG to generate the corresponding PPG, and no additional effort was required for annotating the generated AF PPG data.

### Limitations

In general, PPG signals can be measured using transmission mode or reflectance mode. In this study, all PPG signals used were acquired from the fingertips of subjects undergoing various surgeries using transmission PPG. Meanwhile, smart-watches and wrist-type wearable heart rate monitors are equipped reflectance PPG [25–27]. As the goal of PPG-based AF detection is to be used for such devices rather than during surgeries, where ECG is readily available, the proposed model may not fit this purpose perfectly, as previous studies have shown that PPG measured at different sites have different morphologies [28]. However, the wide subject-to-subject variance and situation-based variance in PPG (such as morphology changes due to shrinking of the capillaries due to anesthesia) may be greater than the regular difference between wrist reflectance PPG and fingertip transmission PPG across subjects. To solve this limitation, the same methodology could be extended using a different dataset which has reflectance PPG. Or an additional transfer function could be made to convert the generated finger PPG signal into a wrist PPG signal.

Second, our model has device and measurement area dependency. Since the waveforms from other device or measured other body area will be slightly different, the morphology of the PPG may be different from our result when using other equipment or from other body area. This problem can be an important issue, considering that the data usually used for AF classification is from a wearable device such as smartwatch. A further study is warranted to validate the proposed method for training AF PPG classification models for use in smartwatches.

Third, there are limits to the objectivity of data labeling. A single cardiologist labeled the AF events in the data used, and this may have introduced biases and errors in AF labels. Lastly, most of the data used in this study were paroxysmal AF types. In the cases where AF causes P-wave depression in ECG rather than arrhythmic heartbeat (which is unseen in PPG), the proposed GAN model may not be able to generate this effect. Therefore, care should be taken in using the data generated using the proposed model, as it does not include all possible cases of AF.

## Conclusion

To the best of our knowledge, this study is the first to use a deep learning model to generate atrial fibrillation PPG from atrial fibrillation ECG. Further, the generated atrial fibrillation PPG was validated to prove that it can be used in the training dataset of the atrial fibrillation classification model. Furthermore, a method to generate a lacking biosignal from another related biosignal with abundant sources was proposed.

To improve the data generation part of this study in the future, the use of ECG-PPG paired data of more diverse conditions as well as patients during anesthesia would be preferred. In addition, with information related to the vascular conditions fed as the input, such as age, to the model during training, the model is expected to create PPG signals that are more realistic. Furthermore, to validate the generated PPG dataset, the broader sense of data practicality can be judged by using it on the classification model that distinguishes not only atrial fibrillation and normal, but also various other arrhythmias.

## Data Availability

Not applicable.
